# Poly(acrylic acid)-Containing Ceria Slurries for Shallow Trench Isolation Chemical Mechanical Polishing: Colloidal Stability, Planarization Efficiency, and Selectivity

**DOI:** 10.3390/polym18151899

**Published:** 2026-08-02

**Authors:** Sohee Hwang, Tao Lyu, Woonjung Kim

**Affiliations:** 1 Department of Chemistry, Hannam University, Daejeon 34430, Republic of Korea; 2 Department of Nanomaterials and Semiconductor, Hannam University, Daejeon 34430, Republic of Korea; lyutao@naver.com

**Keywords:** poly(acrylic acid), polymeric dispersant, ceria nanoparticle, organic–inorganic hybrid slurry, chemical mechanical polishing (CMP), material removal rate (MRR), selectivity

## Abstract

This study reports poly(acrylic acid) (PAA)-containing ceria slurries for shallow trench isolation (STI) chemical mechanical polishing (CMP). HNU15 ceria nanoparticles were prepared by precipitation at room temperature. PAA was synthesized by aqueous free-radical polymerization, characterized by gel permeation chromatography and Fourier-transform infrared spectroscopy, and used as the polymeric dispersant in both HNU15 and commercial HC10 slurries. The primary-particle sizes determined by TEM were 12.2 ± 1.5 nm for HNU15 and 14.6 ± 1.4 nm for HC10, whereas the crystallite sizes calculated from XRD were 10.2 nm and 8.7 nm, respectively. HNU15 showed a higher BET surface area and Ce^3+^ fraction than HC10, indicating measurable differences in textural properties and surface chemical states. After 5 h of milling, the HNU15 and HC10 slurries exhibited DLS d_50_ values of 111 nm and 122 nm and zeta potentials of −53.60 mV and −49.40 mV, respectively. Both slurries maintained generally stable colloidal properties during four weeks of storage at 25 °C and 60 °C. Under the laboratory CMP conditions, HNU15 slurry exhibited an HDP-SiO_2_ removal rate of 114.4 Å min^−1^, an HDP-SiO_2_-to-Si_3_N_4_ selectivity of 8.0, and lower post-polishing roughness than HC10. These results support the use of the PAA-containing HNU15 slurry for STI CMP applications.

## 1. Introduction

Nanomaterials have attracted considerable interest because their physical and chemical properties can differ substantially from those of bulk materials. Among them, ceria-based nanomaterials have been investigated for applications in energy conversion, sensing, biological systems, and surface polishing [1,2,3,4,5,6,7]. In CMP, ceria abrasives are particularly attractive because they can provide effective oxide removal, favorable post-polishing surface quality, and oxide-to-nitride selectivity under appropriately designed slurry conditions.

**Figure 1:**
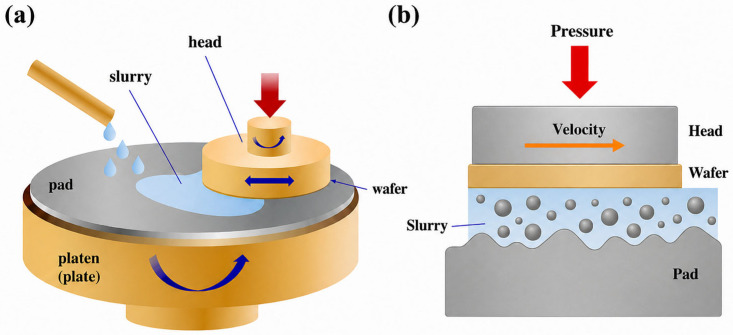
Schematic diagram of the CMP principle: (**a**) overall equipment configuration and (**b**) enlarged cross-sectional view of the polishing interface.

CMP integrates chemical etching and mechanical abrasion to achieve global surface flatness. As illustrated in Figure 1, this synergistic process involves the relative motion of a wafer against a polishing pad under controlled downward pressure, facilitated by the chemical action of the slurry. CMP consumables include slurries, polishing pads, and diamond conditioners. The fundamental mechanics of CMP involve complex lubrication and abrasive behaviors occurring at the interface of the wafer, pad, and slurry film. Maintaining this environment requires the use of a diamond conditioner, which mitigates pad glazing and deformation. By refreshing the pad surface, the conditioner ensures that polishing residues do not clog the pores, thereby sustaining a stable MRR [8].

As device dimensions decrease and surface-quality requirements become more stringent, controlling the microstructure and polishing behavior of ceria abrasives has become increasingly important [9]. In particular, ceria can accommodate changes in oxygen stoichiometry while retaining its fluorite structure [10]. Its Ce^3+^/Ce^4+^ redox chemistry is therefore relevant to surface interactions in ceria-based CMP systems [11].

**Figure 2:**
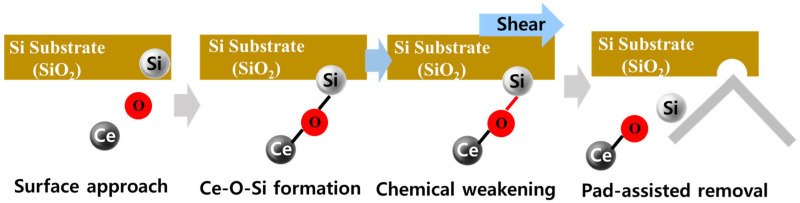
Schematic illustration of the proposed “chemical-tooth” mechanism, in which possible Ce–O–Si interfacial interactions weaken the SiO_2_ surface and pad-induced shear assists the removal of reacted surface material. The gray arrows indicate the progression of the proposed mechanistic steps, the blue arrow indicates shear, and the gray element represents mechanical contact by the polishing pad.

Ceria nanoparticles are widely used as abrasives for SiO_2_ removal in STI CMP, and previous studies have discussed the possible roles of ceria surface states and oxygen exchange in ceria–silica interactions [11,12]. STI is a critical technique for electrically isolating active regions by etching trenches into the silicon substrate. These trenches are subsequently backfilled with insulating dielectrics, such as silicon dioxide, silicon oxynitride, or silicon carbonitride [7]. The proposed “chemical-tooth” mechanism for SiO_2_ removal by ceria abrasives is illustrated in Figure 2.

Cook proposed the “chemical-tooth” model to describe the strong affinity of ceria for hydrated silica surfaces [13]. In aqueous media, surface silanol groups can undergo deprotonation, producing negatively charged silicate species. The literature studies have proposed that hydroxylated ceria and silica surfaces may form interfacial Ce–O–Si species, after which mechanical shear assists the removal of reacted surface material [11,13]. This model provides a useful framework for discussing ceria-based oxide polishing, although individual interfacial steps depend on the slurry composition and polishing conditions. In addition to the abrasive, polymers and other slurry additives can influence dispersion, surface interactions, selectivity, and post-CMP defectivity [7].

Successful CMP depends on multiple factors, including material removal rate, selectivity, defectivity, and post-polishing surface quality. Previous studies have reported associations among ceria surface oxidation states, ceria–silica interactions, and oxide removal behavior [14,15,16,17,18,19,20,21]. In particular, variations in the Ce^3+^/Ce^4+^ ratio have been discussed as one factor that may influence ceria surface reactivity. However, CMP performance also depends on particle size, aggregation state, slurry composition, polishing pressure, pad condition, and other formulation and process variables.

During ceria-based CMP, chemical interactions at the ceria–silica interface weaken the surface layer, while mechanical shear from the polishing pad assists the removal of the reacted material [22]. Repetition of these chemical and mechanical processes contributes to progressive surface planarization. Consequently, colloidal ceria abrasives with controlled particle size and limited aggregation are desirable for achieving consistent oxide removal and surface quality. Polymers and other slurry additives can further influence particle dispersion, interfacial interactions, and oxide-to-nitride selectivity [7].

Numerous methods have been reported for the synthesis of nanoscale ceria, including thermal decomposition [23,24,25,26,27,28], reverse-micelle synthesis [29,30,31], sol–gel processing [32], microemulsion methods [33], sonochemical synthesis [30], and homogeneous precipitation [34,35,36,37,38]. Despite this progress, controlling particle size, morphology, and aggregation while maintaining a practical synthesis procedure remains challenging. Precipitation methods have attracted particular attention because of their relatively simple experimental configuration, potential scalability, and low processing cost. Wang et al. synthesized ceria abrasives using cerium nitrate and ammonia as the cerium precursor and precipitant, respectively [22]. Zhou et al. prepared CeO_2_ nanoparticles of approximately 4 nm using cerium nitrate and ammonium hydroxide [34]. Chen synthesized ceria nanoparticles from cerium nitrate and hexamethylenetetramine [36], whereas Li et al. employed ammonium carbonate and diethylamine as precipitation agents [37]. Additional precipitation-based approaches have also been reported. Yamashita et al. used cerium chloride and sodium hydroxide in the presence of hydrogen peroxide over a pH range of 6–12 [39]. Uekawa et al. synthesized ceria particles of 7–9 nm using cerium nitrate in a polyethylene glycol medium [38]. Chen et al. further demonstrated that ceria particle characteristics were sensitive to reaction temperature and oxygen concentration in the O_2_/N_2_ atmosphere [40]. Previous studies on precipitation-derived ceria have examined the effects of cerium precursors, ligands, additives, reaction media, temperature, and atmosphere on particle characteristics and CMP behavior. Cerium nitrate and ammonium hydroxide are frequently used as the precursor and precipitant, respectively, in precipitation-based ceria synthesis. Depending on the synthesis and post-treatment conditions, colloidal ceria can exhibit different morphological and particle-size characteristics. Colloidal ceria behavior is also relevant to particle contamination and cleaning after oxide CMP [41]. These considerations have motivated continued interest in colloidal ceria slurry design.

Ceria particle size can influence CMP performance parameters such as material removal rate, surface roughness, selectivity, and defectivity. Larger abrasive particles may increase mechanical contact and removal under some polishing conditions, whereas other studies have reported different or non-monotonic trends depending on particle synthesis, aggregation state, slurry composition, and process conditions [42]. Therefore, particle size should be considered together with the overall slurry formulation rather than as an independent predictor of CMP performance.

Poly(acrylic acid) (PAA) was selected as the polymeric dispersant because its carboxyl groups can interact with metal-oxide surfaces and contribute electrostatic and steric resistance to particle approach in aqueous media [43,44]. In this study, PAA was synthesized by aqueous free-radical polymerization and characterized by GPC and FT-IR. The synthesized PAA was subsequently added at the same fixed loading to slurries containing room-temperature-precipitated HNU15 ceria and commercial HC10 ceria. Both PAA-containing slurries were evaluated through milling-dependent measurements of pH, DLS d_50_, and zeta potential, four-week storage-stability tests at 25 °C and 60 °C, and CMP performance evaluation. This design provides a framework to discuss the relationships among polymer characteristics, ceria–water interfacial behavior, colloidal properties, storage stability, and polishing performance at the slurry level.

## 2. Materials and Methods

### 2.1. Materials

Cerium nitrate hexahydrate (99.8%, Ce(NO_3_)_3_·6H_2_O; Kanto Chemical Co., Inc., Tokyo, Japan), ammonium hydroxide (28–30 wt% NH_4_OH; Junsei Chemical Co., Ltd., Tokyo, Japan), and deionized water (18.2 MΩ cm at 25 °C; Aquapuri 541, Youngin Chromass, Anyang, Republic of Korea) were used for the synthesis of HNU15 ceria nanoparticles. Acrylic acid (AA; 99%, containing 200 ppm monomethyl ether hydroquinone as an inhibitor; Sigma-Aldrich, St. Louis, MO, USA) and ammonium persulfate (APS; ≥98.0%; Alfa Aesar, Haverhill, MA, USA) were used for PAA synthesis. High-purity nitrogen (99.999%; Deokyang Co., Ltd., Ulsan, Republic of Korea) was used during the polymerization process. Commercial HC10 ceria powder (Solvay, Brussels, Belgium) was used as the comparison abrasive. A commercial polymeric dispersant was used solely as a qualitative reference for FT-IR comparison and was not used in the preparation of either CMP slurry. The synthesized PAA was the only polymeric dispersant used in both the HNU15 and HC10 slurries at the same fixed loading. All chemicals were used as received without further purification.

### 2.2. Preparation of PAA

**Figure 3:**
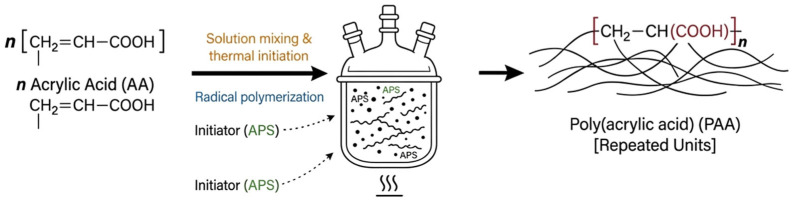
Schematic illustration of PAA synthesis via aqueous free-radical polymerization initiated by APS. The solid arrows indicate the main polymerization sequence, whereas the dashed arrows indicate the addition of APS as the radical initiator.

PAA was synthesized by aqueous free-radical polymerization. A 9.25 M acrylic acid solution was prepared in 300 mL of deionized water in a three-neck round-bottom flask. The solution was mechanically stirred and purged with nitrogen for 30 min to remove dissolved oxygen. An ammonium persulfate solution (APS; 0.35 M in 50 mL of deionized water) was then added as the initiator. The reaction mixture was stirred at 80 °C for 4 h. After cooling to room temperature, the resulting PAA solution was neutralized with 10 wt% NH_4_OH and used for subsequent slurry preparation. The PAA synthesis procedure is schematically illustrated in Figure 3.

### 2.3. Preparation of HNU15 Ceria Nanoparticles

Ceria nanoparticles, denoted as HNU15, were synthesized by a room-temperature liquid-phase precipitation method. Cerium (III) nitrate hexahydrate was dissolved in 100 mL of deionized water to prepare a 0.1 M Ce(NO_3_)_3_·6H_2_O solution, which was magnetically stirred at 500 rpm. Subsequently, a 10 wt% NH_4_OH solution was added dropwise at 1.0 mL min^−1^ until the pH reached 9.0 to 10.0. The solution pH was monitored throughout the addition. A pale cerium-containing precipitate was formed and collected by filtration. The precipitate was washed several times with deionized water and ethanol until the filtrate reached approximately pH 7.0. The collected precipitate was dried under vacuum at 110 °C for 24 h and subsequently calcined in air at 400 °C for 4 h at a heating rate of 5 °C min^−1^ to obtain crystalline CeO_2_ nanoparticles. The precipitation and calcination processes can be represented by the following simplified reactions:

Ce(NO_3_)_3_ + 3 NH_4_OH → Ce(OH)_3_↓ + 3 NH_4_NO_3_

4 Ce(OH)_3_ + O_2_ → 4 CeO_2_ + 6 H_2_O

### 2.4. Preparation of PAA-Containing CMP Slurries

PAA-containing CMP slurries were prepared using the synthesized HNU15 and commercial HC10 ceria powders under identical formulation and milling conditions. The synthesized PAA was used as a polymeric dispersant in both slurries at the same fixed loading. Each initial milling slurry had a total mass of 600 g and contained 180 g of CeO_2_ powder (30 wt%) and 4.50 g of PAA solids (0.75 wt% of the total slurry and 2.5 wt% relative to the CeO_2_ powder), with DI water comprising the remaining mass.

The slurries were premixed and wet-milled at 500 rpm for 5 h using a Feather-Crusher LCD Display system (Daihan Scientific Co., Ltd., Wonju, Republic of Korea) with zirconia beads 0.2–0.8 mm in diameter. Aliquots were collected before milling and after 1, 2, 3, 4, and 5 h for pH, DLS d_50_, and zeta potential measurements. After 5 h of milling, the slurries were passed through a 10 μm nylon mesh to remove unusually large coarse particles or agglomerates. The solid content after filtration was determined gravimetrically by drying a known mass of slurry to constant mass, and each slurry was diluted with deionized water to a final CeO_2_ concentration of 4.05 wt% before CMP testing.

### 2.5. Characterization and CMP Performance Evaluation

#### 2.5.1. Transmission Electron Microscopy and High-Resolution Transmission Electron Microscopy

Transmission electron microscopy (TEM; JEM-2100F, JEOL Ltd., Tokyo, Japan) was used to examine the morphology and primary-particle size of the prepared HNU15 and commercial HC10 ceria powders. High-resolution transmission electron microscopy (HRTEM) was performed using a Talos F200X field-emission transmission electron microscope (Thermo Fisher Scientific/FEI, Waltham, MA, USA) operated at an accelerating voltage of 200 kV at the KAIST Analysis Center for Research Advancement (KARA). Primary-particle sizes were measured from representative TEM images using ImageJ software (version 1.54h; National Institutes of Health, Bethesda, MD, USA). A total of 100 particles were analyzed for each sample, and the results are reported as the mean ± standard deviation. Selected-area electron diffraction (SAED) patterns were obtained for qualitative identification of the crystalline phase.

#### 2.5.2. Scanning Electron Microscopy and Energy-Dispersive X-Ray Spectroscopy

The morphology of the prepared HNU15 and commercial HC10 ceria powders was examined using a JSM-7610F Plus field-emission scanning electron microscope (JEOL Ltd., Tokyo, Japan). Energy-dispersive X-ray spectroscopy (EDX) was performed using the EDX system attached to the microscope to confirm the presence of cerium and oxygen in the samples.

#### 2.5.3. X-Ray Diffraction

X-ray diffraction (XRD) patterns of the prepared HNU15 and commercial HC10 ceria powders were recorded using an X’Pert PRO diffractometer (PANalytical, Almelo, The Netherlands) with Cu Kα radiation (λ = 1.5418 Å) in a Bragg–Brentano configuration. The crystalline phases were identified by comparison with the reference pattern for cubic fluorite CeO_2_ (PDF No. 43-1002). The average crystallite size of each sample was calculated from the (111) reflection using the Scherrer equation [45]. The integrated areas of the indexed reflections were obtained by peak fitting, and the relative contribution of each reflection was calculated by normalizing its integrated area to the sum of the integrated areas of all indexed reflections.

#### 2.5.4. N_2_ Adsorption–Desorption Analysis

N_2_ adsorption–desorption isotherms of the prepared HNU15 and commercial HC10 ceria powders were measured using a NOVA 2200e surface-area and pore-size analyzer (Quantachrome Instruments, Boynton Beach, FL, USA). Before analysis, the samples were degassed under dynamic vacuum at 200 °C for 4 h. The adsorption–desorption measurements were performed at liquid-nitrogen temperature. The specific surface area was calculated using the multipoint Brunauer–Emmett–Teller (BET) method, and the average pore size was obtained from the corresponding pore-size analysis.

#### 2.5.5. X-Ray Photoelectron Spectroscopy

The prepared HNU15 and commercial HC10 ceria powders were analyzed by X-ray photoelectron spectroscopy (XPS) using an ESCALAB 250Xi instrument (Thermo Fisher Scientific, Waltham, MA, USA) with monochromatic Al Kα radiation at 1486.6 eV. All binding energies were calibrated to the C 1s peak at 284.8 eV. High-resolution Ce 3d and O 1s spectra were analyzed by peak deconvolution. The Ce^3+^ fraction was calculated from the integrated areas of the fitted Ce^3+^ components relative to the total fitted Ce 3d peak area.

#### 2.5.6. Gel Permeation Chromatography

The molecular-weight distribution of the synthesized PAA was determined by gel permeation chromatography (GPC; EcoSEC HLC-8420 GPC, Tosoh, Tokyo, Japan) equipped with a refractive-index detector. The PAA sample was dissolved in the mobile phase at a concentration of 15 mg mL^−1^ and filtered through a 0.22 μm nylon syringe filter before injection. An aqueous 0.1 M NaNO_3_ solution was used as the mobile phase at a flow rate of 1.0 mL min^−1^. Chromatographic separation was performed at 40 °C using a TSKgel guard PWXL column, two TSKgel GMPWXL columns, and one TSKgel G2500PWXL column (Tosoh, Tokyo, Japan) connected in series. Poly(ethylene glycol)/poly(ethylene oxide) standards were used for molecular-weight calibration, and the injection volume was 50 μL. The data were processed using EcoSEC Elite-WS software (Tosoh, Tokyo, Japan).

#### 2.5.7. Fourier-Transform Infrared Spectroscopy

Fourier-transform infrared (FT-IR) spectra of the synthesized PAA and the commercial polymeric dispersant used as a qualitative reference were obtained using a Nicolet Summit X FT-IR spectrometer (Thermo Fisher Scientific, Waltham, MA, USA). The spectra were used to compare their characteristic functional groups and to evaluate whether the synthesized polymer exhibited the expected PAA-related absorption bands.

#### 2.5.8. pH, DLS, and Zeta Potential Measurements

The pH of the PAA-containing HNU15 and HC10 slurries was measured using an Orion Star A215 pH meter (Thermo Fisher Scientific, Waltham, MA, USA). Dynamic light scattering measurements were performed using an ELS-2000 analyzer (Otsuka Electronics, Osaka, Japan), and the median hydrodynamic diameter was reported as DLS d_50_. Zeta potential was measured using a Nanotrac Wave II analyzer (Microtrac, Osaka, Japan). The same measurement procedures were applied to both slurry formulations throughout the milling and storage-stability evaluations.

#### 2.5.9. Storage Stability Evaluation

After 5 h of ball milling, the PAA-containing HNU15 and HC10 slurries were stored at 25 °C and 60 °C for four weeks. The pH, DLS d_50_, and zeta potential were measured at week 0 and at weekly intervals thereafter for four weeks. The week 0 values correspond to measurements performed immediately after completion of the 5 h milling process. All measurements were conducted using the instruments and procedures described in Section 2.5.8.

#### 2.5.10. CMP Evaluation

Polishing experiments were performed using a POLI-500 laboratory CMP system (G&P Technology, Inc., Busan, Republic of Korea). An IC1000 polyurethane polishing pad (DuPont, Wilmington, DE, USA) and a diamond-grit conditioner (3M Company, St. Paul, MN, USA) were used. The platen speed was 300 rpm, the carrier speed was 150 rpm, the applied pressure was 4 psi, the slurry flow rate was 28.5 mL min^−1^, and the polishing time was 60 s. The same polishing conditions, pad, and conditioning procedure were used for the PAA-containing HNU15 and HC10 slurries.

High-density plasma silicon dioxide (HDP-SiO_2_) and silicon nitride (Si_3_N_4_) films deposited on silicon wafers were used as the polishing substrates. Film thicknesses before and after polishing were measured at 10 locations distributed radially from the wafer center toward the edge using an optical reflectometer (EH-OEM, Ellitop Scientific Co., Ltd., Beijing, China). The local material removal rate at each measurement location was calculated according to Equation (1):

(1) $$MRRi=\frac{d_{0,i}-d_{1,i}}{t}\times 10$$

where *d*_0,*i*_ and *d*_1,*i*_ are the film thicknesses in nanometers before and after polishing at measurement location *i*, respectively; *t* is the polishing time in minutes; and the factor of 10 converts nm min^−1^ to Å min^−1^. The average MRR was calculated as the arithmetic mean of the local MRR values obtained from the 10 measurement locations. The HDP-SiO_2_-to-Si_3_N_4_ selectivity was calculated as the average HDP-SiO_2_ MRR divided by the average Si_3_N_4_ MRR.

Within-wafer non-uniformity (WIWNU) was calculated from the maximum, minimum, and average local MRR values according to Equation (2) [46]:

(2) $$WIWNU(\%)=\frac{{MRR}_{max}-{MRR}_{min}}{{2MRR}_{avg}}\times 100$$

where MRRmax, MRRmin, and MRRavg represent the maximum, minimum, and average MRR values obtained from the 10 measurement locations, respectively.

#### 2.5.11. Atomic Force Microscopy

Post-polishing surface topography was examined using an nGauge atomic force microscope (AFM; ICSPI Corp., Kitchener, ON, Canada) operated in intermittent-contact mode under ambient conditions. Topographic images were acquired over an area of 2.0 × 2.0 μm^2^ using nGauge Research software (version 1.2.7.5; ICSPI Corp., Kitchener, ON, Canada). The acquired height images were analyzed using Gwyddion software (version 2.67; Czech Metrology Institute, Brno, Czech Republic), and the root-mean-square roughness (Rq) was determined over the entire scan area.

## 3. Results and Discussion

### 3.1. Morphology and Structure of the Ceria Powders

**Figure 4:**
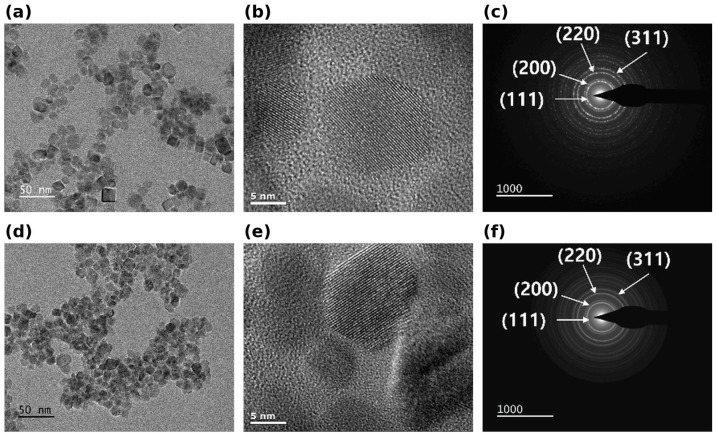
Representative TEM images (**a**,**d**), HRTEM images (**b**,**e**), and SAED patterns (**c**,**f**) of HNU15 (**a**–**c**) and HC10 (**d**–**f**). The SAED patterns were used for qualitative phase identification.

**Figure 5:**
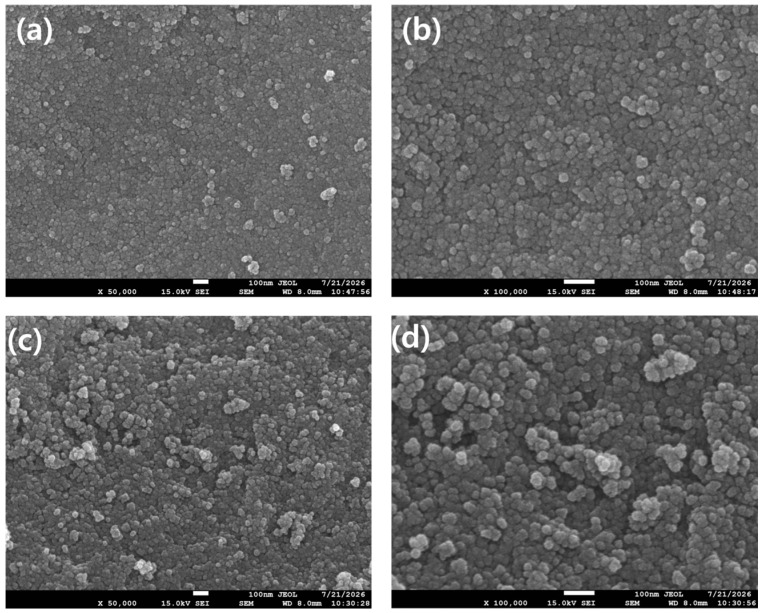
Representative SEM images of prepared HNU15 (**a**,**b**) and commercial HC10 (**c**,**d**). The scale bar in each panel represents 100 nm.

Representative TEM, HRTEM, SAED, and SEM images were used to examine the morphology and crystal structure of the prepared HNU15 and commercial HC10 ceria powders, as shown in Figure 4 and Figure 5. Both powders consisted of approximately rounded nanoscale primary particles. Quantitative TEM analysis using ImageJ determined primary-particle sizes of 12.2 ± 1.5 nm for HNU15 and 14.6 ± 1.4 nm for HC10 based on 100 particles analyzed for each sample. Particle shape was evaluated qualitatively, whereas primary-particle size was evaluated quantitatively from the TEM images. Dry-state particle associations were visible in both samples; however, these observations are not used to represent the dispersion state of the final aqueous slurries after PAA addition and milling. Previous studies have reported that abrasive particle size and morphology can influence CMP contact behavior and surface quality [47,48,49], but the present TEM images alone do not establish a direct relationship with MRR or post-polishing defects.

Both samples exhibited SAED rings corresponding to the (111), (200), (220), and (311) planes of cubic fluorite CeO_2_, consistent with the phase assignment obtained by XRD. The TEM-derived primary-particle sizes (*d*_TEM_) differed slightly from the XRD-derived crystallite sizes (*d*_XRD_), which is reasonable because TEM evaluates individual particles in selected image regions, whereas XRD provides a bulk-averaged estimate of coherent crystalline-domain size.

The SEM images showed fine particulate morphologies and dry-powder agglomerates in both samples. EDX analysis detected cerium and oxygen within the measurement sensitivity. Because dry-powder SEM does not represent the aqueous state after PAA addition and milling, Figure 5 is used for morphological comparison rather than for ranking the dispersion stability of the final slurries.

### 3.2. X-Ray Diffraction Analysis of the Ceria Powders

**Figure 6:**
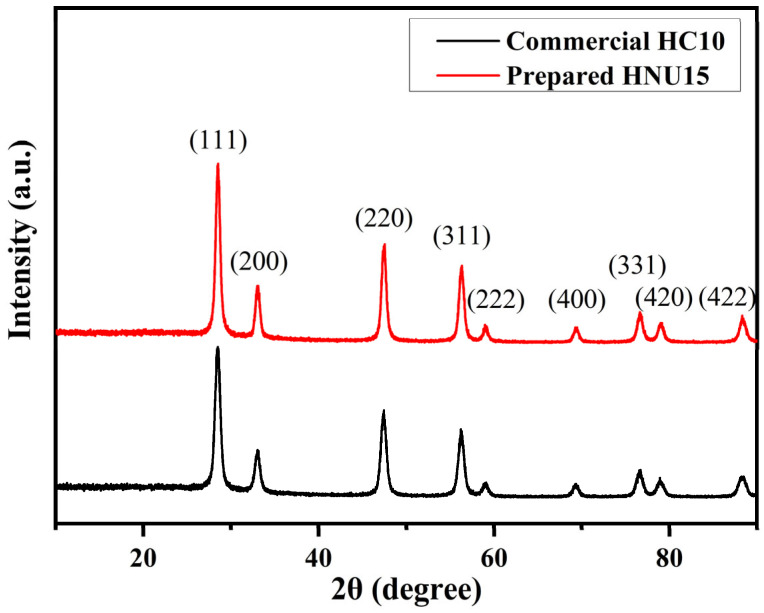
X-ray diffraction patterns of prepared HNU15 and commercial HC10 ceria powders. The indexed reflections correspond to cubic fluorite CeO_2_ (PDF No. 43-1002).

As shown in Figure 6, the diffraction peaks at approximately 28°, 33°, 47°, and 56° were assigned to the (111), (200), (220), and (311) planes, respectively, of cubic fluorite CeO_2_ (space group Fm−3m; PDF No. 43-1002). The crystallite sizes calculated from the (111) reflection using the Scherrer equation were 10.2 nm for HNU15 and 8.7 nm for HC10. The broadened diffraction peaks observed for both samples were consistent with their nanoscale coherent crystalline-domain sizes. The TEM-derived primary-particle sizes (*d*_TEM_) differed slightly from the XRD-derived crystallite sizes (*d*_XRD_), because the two techniques characterize different structural length scales.

The integrated area of each indexed reflection was obtained by peak fitting. The relative integrated intensity of each reflection was calculated by normalizing its integrated area to the sum of the integrated areas of all indexed reflections, as expressed in Equation (3). The normalized relative integrated intensities of the indexed reflections are summarized in Table 1.

(3) $$P_{\left(hkl\right)}(\%)=\frac{I_{\left(hkl\right)}}{\sum I_{\left(hkl\right)}}\times 100$$

where I(*hkl*) is the integrated intensity (peak area) of the corresponding (hkl) reflection. The percentages were calculated by normalizing the integrated peak area of each indexed reflection to the sum of the integrated areas of all listed reflections.

### 3.3. N_2_ Adsorption–Desorption Analysis of the Ceria Powders

**Figure 7:**
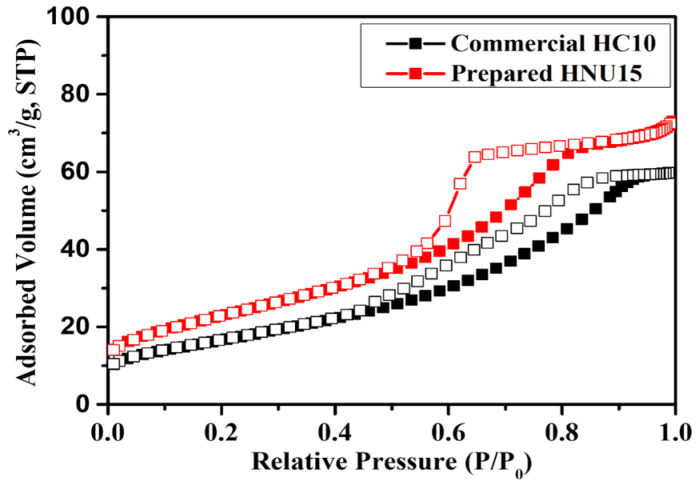
N_2_ adsorption–desorption isotherms of prepared HNU15 and commercial HC10 powders. Filled square markers indicate the adsorption branch, whereas open square markers indicate the desorption branch.

The textural properties of HNU15 and HC10 were evaluated using N_2_ adsorption–desorption isotherms, as shown in Figure 7. HNU15 exhibited a BET surface area of 83.2 m^2^ g^−1^ and an average pore size of 5.42 nm, whereas HC10 exhibited a BET surface area of 60.2 m^2^ g^−1^ and an average pore size of 6.14 nm. Both samples exhibited Type IV adsorption–desorption behavior with hysteresis at intermediate-to-high relative pressures, consistent with mesopore-range adsorption behavior and/or interparticle voids formed by aggregated nanoparticles [50,51]. Differences in the hysteresis profiles indicate differences in the textural or interparticle-packing characteristics of the two powders.

Table 2 summarizes the TEM-derived primary-particle sizes, XRD-derived crystallite sizes, BET surface areas, and average pore sizes of HNU15 and HC10. The BET measurements demonstrate a larger specific surface area for HNU15; however, the measured surface area does not directly quantify the density of chemically active polishing sites.

### 3.4. X-Ray Photoelectron Spectroscopy of the Ceria Powders

**Figure 8:**
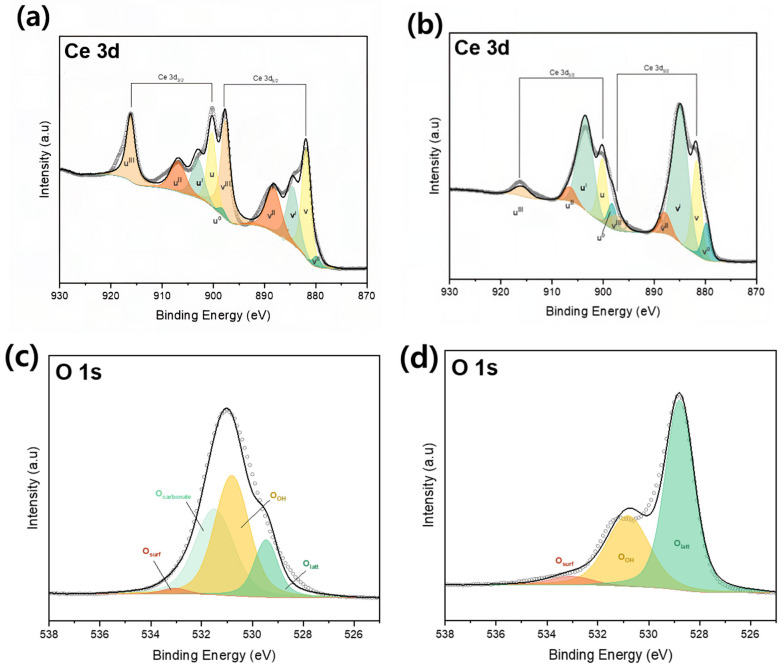
High-resolution XPS spectra and fitted components of the Ce 3d regions (**a**,**b**) and O 1s regions (**c**,**d**) for commercial HC10 (**a**,**c**) and prepared HNU15 (**b**,**d**). Open symbols represent the experimental data, solid black curves represent the overall fitted spectra, and the colored filled curves represent the individual fitted components. The Ce 3d components assigned to Ce^4+^ are v, v″, v‴, u, u″, and u‴, whereas v_0_, v′, u_0_, and u′ are assigned to Ce^3+^. The labels in panels (**c**,**d**) identify the corresponding fitted O 1s components.

X-ray photoelectron spectroscopy (XPS) was used to examine the surface chemical states of the prepared HNU15 and commercial HC10 ceria powders. Figure 8 presents the fitted high-resolution Ce 3d and O 1s spectra of both samples. The Ce 3d spectrum was deconvoluted into the Ce 3d_5_/_2_ region, denoted as v, and the Ce 3d_3_/_2_ region, denoted as u. The v, v″, v‴, u, u″, and u‴ components were assigned to Ce^4+^, whereas the v_0_, v′, u_0_, and u′ components were assigned to Ce^3+^ [52,53]. The Ce^3+^ fraction was calculated from the integrated areas of the fitted Ce^3+^ components relative to the total fitted Ce 3d peak area, as expressed in Equation (4).

(4) $${Ce}^{3+}fraction\;(\%)=\frac{A\left(v_{0}\right)+A{(v}^{\prime })+A\left(u_{0}\right)+{A(u}^{\prime })}{A(total\;Ce\;3d)}\times 100$$

where A represents the integrated area of the corresponding fitted Ce 3d component.

The Ce^3+^ fractions calculated from the fitted Ce 3d spectra were 22.1% for HNU15 and 17.7% for HC10, with corresponding Ce^4+^ fractions of 77.9% and 82.3%, respectively. These results demonstrate a measurable difference in the cerium surface chemical states of the two powders. The higher Ce^3+^ fraction of HNU15 is consistent with a greater relative contribution of Ce^3+^-associated surface states. However, the Ce^3+^ fraction is not interpreted as a direct quantitative measurement of oxygen-vacancy concentration.

The fitted O 1s spectra contained a lower-binding-energy component assigned to lattice oxygen and a higher-binding-energy component associated with surface or adsorbed oxygen species. The higher-binding-energy component was located at 533.05 eV for HNU15 and 533.20 eV for HC10. The Ce oxidation-state fractions and selected O 1s binding energies are summarized in Table 3.

Previous studies have discussed associations between Ce^3+^-related surface states and ceria–silica interfacial interactions during CMP [15,16,17,18,19,20,21,22,54]. Accordingly, the higher Ce^3+^ fraction observed for HNU15 may be relevant to differences in interfacial behavior under the evaluated CMP conditions. Nevertheless, the present ex situ XPS results are interpreted as evidence of differences in surface chemical states rather than as direct proof of Ce–O–Si bond formation or the CMP reaction mechanism.

### 3.5. Characterization of the Synthesized PAA Dispersant

**Figure 9:**
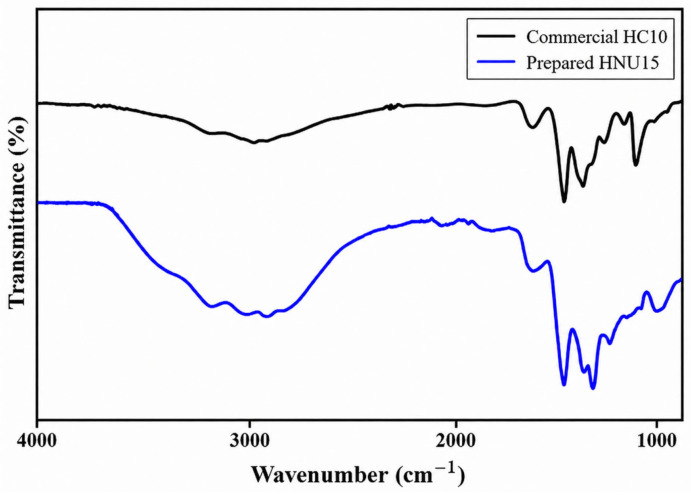
FT-IR spectra of the synthesized PAA dispersant (blue) and the commercial polymeric dispersant used as a qualitative reference (black).

To characterize the synthesized PAA dispersant, the FT-IR spectra of the synthesized PAA and the commercial polymeric dispersant used as a qualitative reference were compared, as shown in Figure 9. The commercial dispersant was included only as a characterization reference, whereas the synthesized PAA was used as the polymeric dispersant in both the HNU15 and HC10 slurries at the same fixed loading.

The synthesized PAA exhibited absorption bands characteristic of PAA-related structures. The broad band at 3000–3600 cm^−1^ was assigned to O–H stretching of carboxylic acid groups and hydrogen-bonded species. The band near 1700 cm^−1^ corresponded to carbonyl C=O stretching, whereas the bands near 1550 and 1400 cm^−1^ were assigned to the asymmetric and symmetric stretching vibrations of carboxylate groups, respectively. These bands support the presence of carboxylic acid and carboxylate functionalities in the synthesized PAA.

GPC analysis determined a number-average molecular weight (Mn) of 11,500 g mol^−1^, a weight-average molecular weight (Mw) of 21,300 g mol^−1^, and a polydispersity index (PDI, Mw/Mn) of 1.85 for the synthesized PAA. The measured PDI indicates a distribution of polymer chain lengths consistent with conventional free-radical polymerization. The molecular-weight characteristics of the synthesized PAA are summarized in Table 4.

### 3.6. Colloidal Properties and Milling Response of PAA-Containing Ceria Slurries

**Figure 10:**
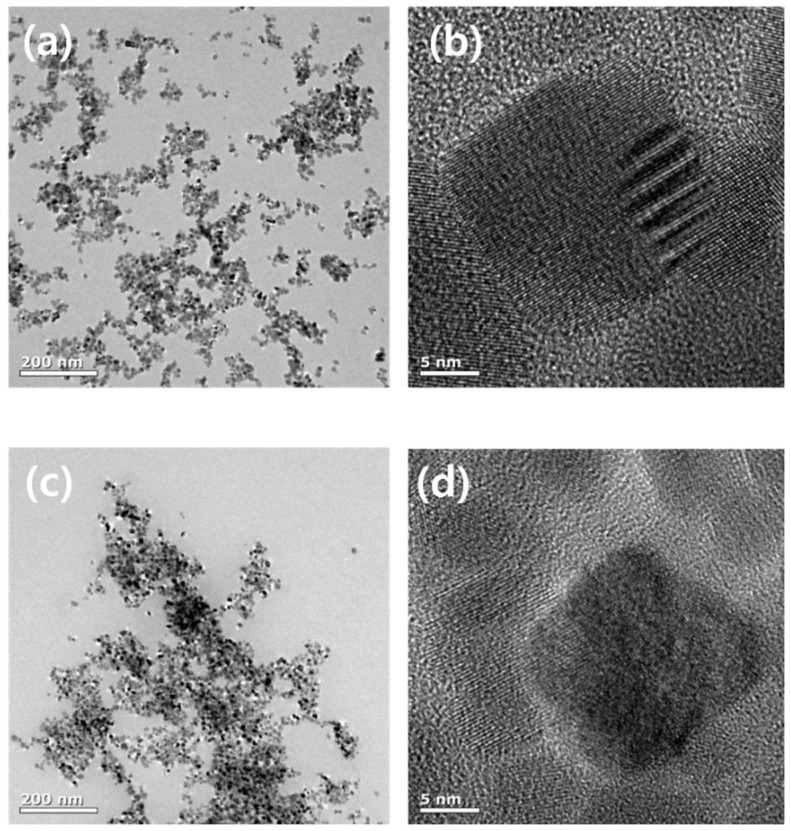
Representative post-milling TEM images of the PAA-containing HNU15 slurry at (**a**) low and (**b**) high magnification and the PAA-containing HC10 slurry at (**c**) low and (**d**) high magnification.

Figure 10 shows representative post-milling TEM images of the PAA-containing HNU15 and HC10 slurries. Both samples retained nanoscale ceria particles, with local particle associations visible in the dried TEM specimens. Because drying during TEM sample preparation can alter particle arrangement, these images are interpreted qualitatively together with the DLS and zeta-potential measurements rather than as quantitative evidence of slurry dispersion or structural degradation.

Table 5 summarizes the milling-dependent pH, DLS d_50_, and zeta-potential values of the two PAA-containing slurries over 5 h of milling. Before milling, the DLS d_50_ values were 155 nm for HNU15 and 160 nm for HC10. After 5 h of milling, the values decreased to 111 nm and 122 nm, respectively. During the same period, the zeta potential changed from −45.55 mV to −53.60 mV for HNU15 and from −43.33 mV to −49.40 mV for HC10, while the pH increased from 8.51 to 9.11 and from 7.52 to 8.43, respectively. Previous studies have shown that PAA molecular weight and concentration can influence ceria interfacial and dispersion behavior [43,44]. In the present study, these measurements characterize the complete PAA-containing slurries. Accordingly, the smaller DLS d_50_ and more negative zeta potential observed for HNU15 after milling are interpreted as slurry-level differences rather than as an isolated quantitative effect of PAA as a polymeric dispersant.

**Figure 11:**
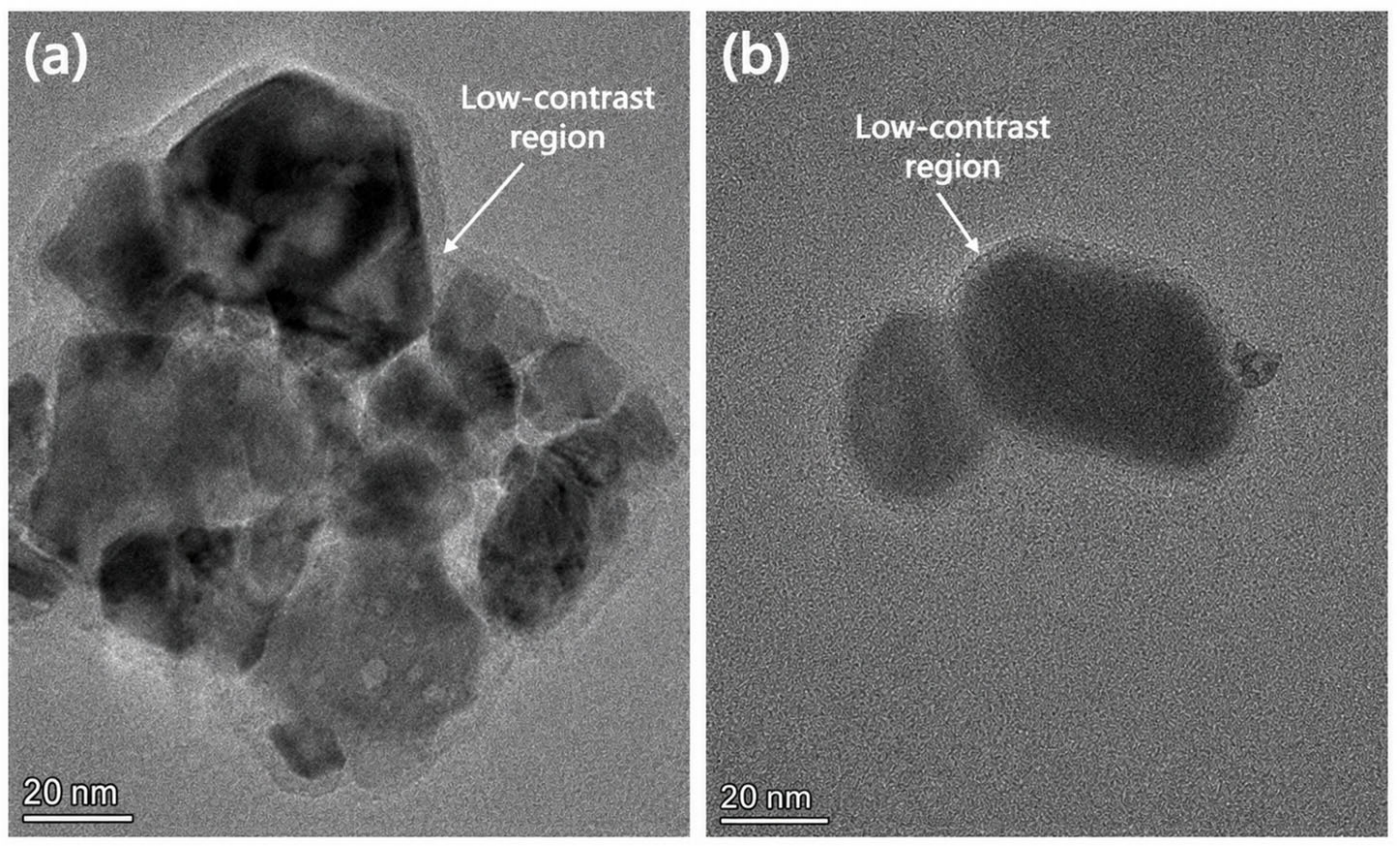
Representative HRTEM images showing local surface contrast around (**a**) PAA-containing commercial HC10 and (**b**) PAA-containing prepared HNU15 particles.

**Figure 12:**
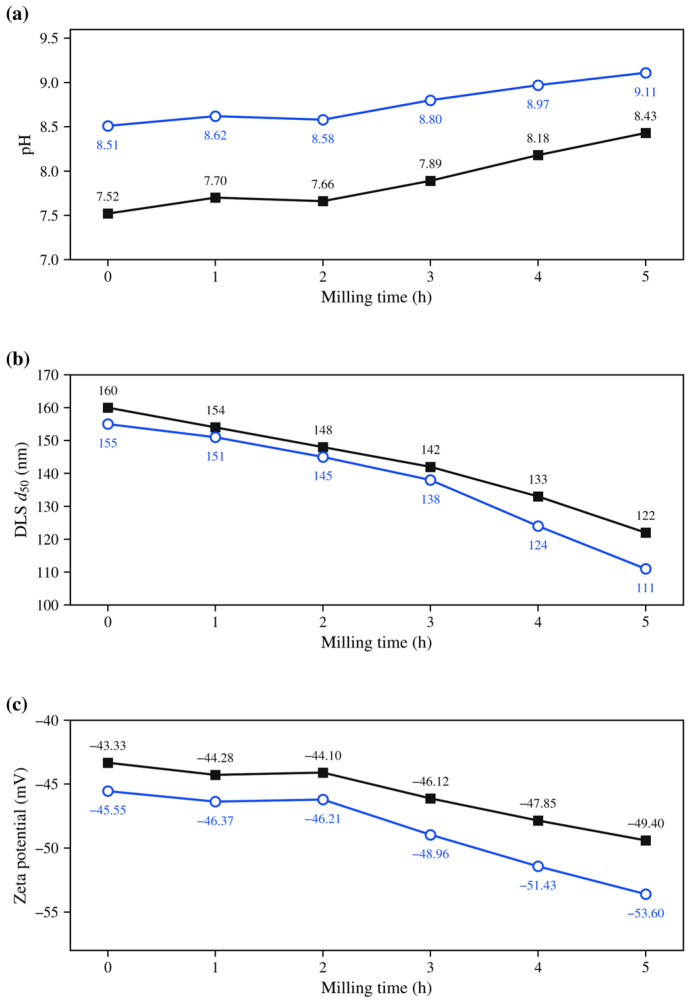
Milling-dependent properties of the PAA-containing HC10 (black) and HNU15 (blue) slurries from 0 to 5 h: (**a**) pH, (**b**) DLS d_50_, and (**c**) zeta potential.

Figure 11 presents representative HRTEM images of particles from the PAA-containing HC10 and HNU15 slurries. Local low-contrast surface regions were observed around selected particles, including an apparent region of approximately 2 nm to 3 nm around selected HNU15 particles. Such contrast may reflect adsorbed interfacial material and/or effects introduced during specimen preparation and imaging; it is not treated as direct chemical identification of a continuous PAA coating. The observations are therefore interpreted qualitatively together with the DLS, zeta-potential, and storage-stability results. The corresponding milling-dependent changes in pH, DLS d_50_, and zeta potential are presented in Figure 12.

### 3.7. CMP Performance of the PAA-Containing Ceria Slurries

**Figure 13:**
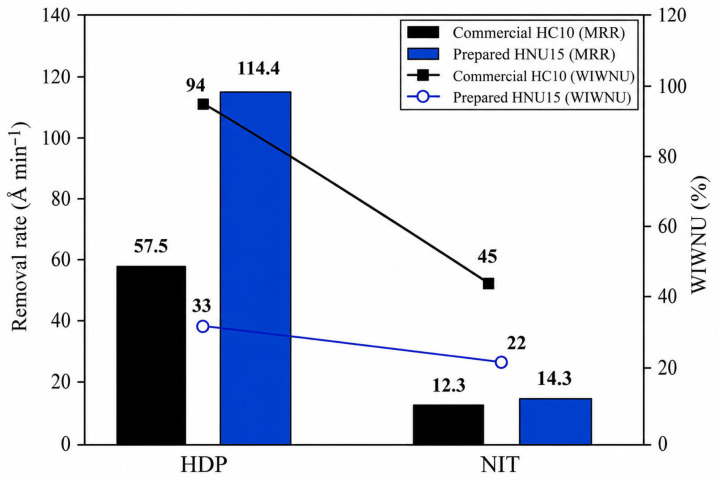
Comparison of material removal rate (MRR; bars, left axis) and within-wafer non-uniformity (WIWNU; lines, right axis) for HDP-SiO_2_ and Si_3_N_4_ films polished with the PAA-containing HNU15 and HC10 slurries. Solid bars and circular markers represent HNU15, whereas hatched bars and square markers represent HC10.

Figure 13 compares the material removal rate (MRR) and within-wafer non-uniformity (WIWNU) obtained with the PAA-containing HNU15 and HC10 slurries. The HNU15 slurry produced MRR values of 114.4 Å min^−1^ for HDP-SiO_2_ and 14.3 Å min^−1^ for Si_3_N_4_, whereas the corresponding values obtained with the HC10 slurry were 57.5 Å min^−1^ and 12.3 Å min^−1^, respectively. These comparisons are presented descriptively because statistical significance was not evaluated using independent replicate polishing runs.

Based on the unrounded MRR values, the HDP-SiO_2_-to-Si_3_N_4_ selectivity was 8.0 for the HNU15 slurry and 4.7 for the HC10 slurry. Thus, the HNU15 slurry exhibited a higher oxide-to-nitride selectivity under the common laboratory polishing conditions. The WIWNU values for the HNU15 slurry were 33% for HDP-SiO_2_ and 22% for Si_3_N_4_, compared with 94% and 45%, respectively, for the HC10 slurry. The lower WIWNU values obtained with the HNU15 slurry indicate a more uniform removal profile across the measured wafer locations under the evaluated conditions. Because both slurries contained the same PAA loading and were tested under identical CMP conditions, the observed differences are interpreted at the slurry level rather than being attributed exclusively to either PAA or a single ceria-powder characteristic.

**Figure 14:**
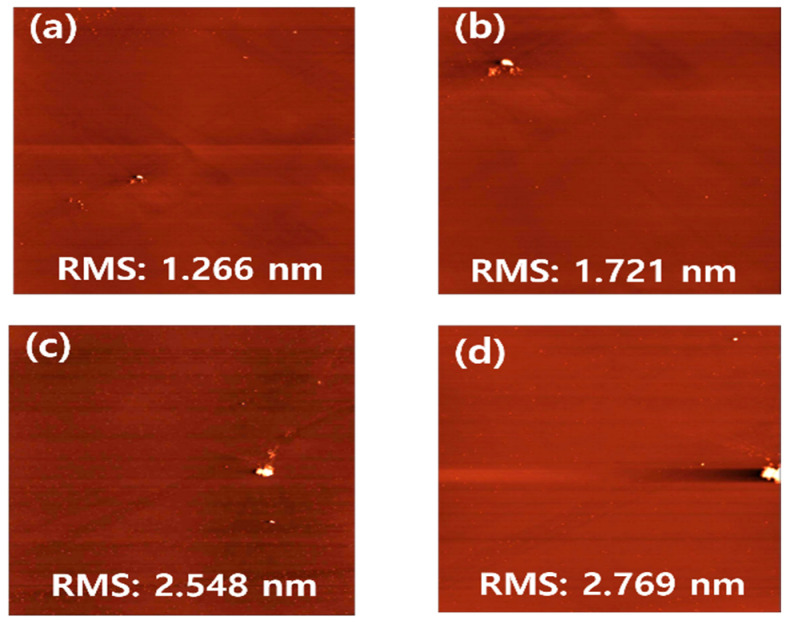
AFM topographic images acquired over an area of 2.0 × 2.0 μm^2^ after polishing HDP-SiO_2_ (**a**,**c**) and Si_3_N_4_ (**b**,**d**) with the PAA-containing HNU15 (**a**,**b**) and HC10 (**c**,**d**) slurries.

Figure 14 presents the post-polishing surface topographies measured by atomic force microscopy. The PAA-containing HNU15 slurry produced root-mean-square (RMS) roughness, Rq, values of 1.266 nm for HDP-SiO_2_ and 1.721 nm for Si_3_N_4_. The corresponding values obtained with the PAA-containing HC10 slurry were 2.548 nm and 2.769 nm, respectively. Scratch-like surface features were also visible in the representative AFM images of the surfaces polished with the HC10 slurry.

The lower post-polishing roughness obtained with the HNU15 slurry is consistent with its smaller post-milling DLS d_50_ and more negative zeta potential. However, these AFM differences should be interpreted as the combined result of the complete slurry system, including ceria-powder characteristics, PAA–particle interfacial interactions, and milling-derived dispersion. They are therefore not assigned to a continuous PAA coating or to any single material property.

### 3.8. Stability of the PAA-Containing Ceria Slurries

**Figure 15:**
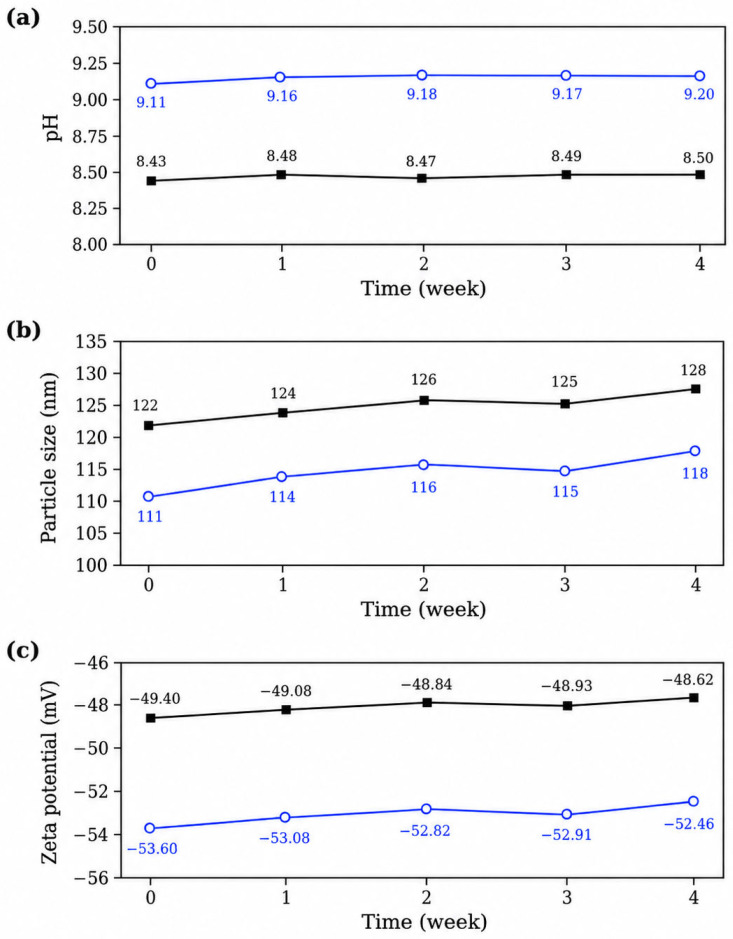
Four-week storage stability of the PAA-containing HNU15 (blue) and HC10 (black) slurries at 25 °C: (**a**) pH, (**b**) DLS d_50_, and (**c**) zeta potential.

**Figure 16:**
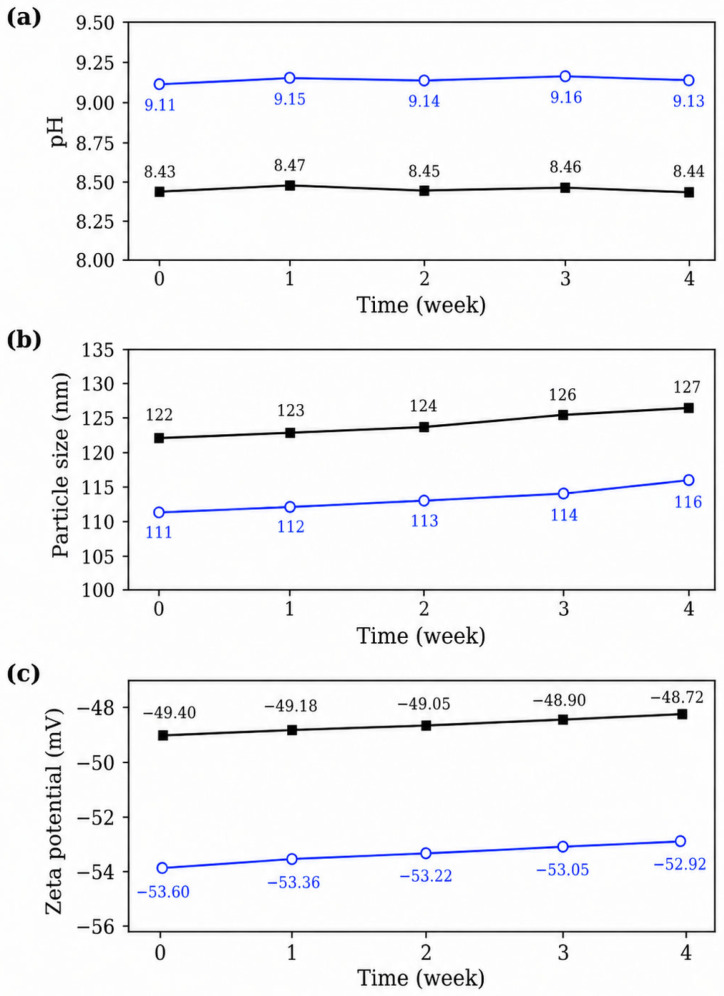
Four-week storage stability of the PAA-containing HNU15 (blue) and HC10 (black) slurries at 60 °C: (**a**) pH, (**b**) DLS d_50_, and (**c**) zeta potential.

To evaluate storage stability, the pH, DLS d_50_, and zeta potential of the PAA-containing HNU15 and HC10 slurries were monitored weekly for four weeks at 25 °C and 60 °C, as shown in Figure 15 and Figure 16. The week 0 values correspond to measurements obtained immediately after completion of the 5 h milling process.

At 25 °C, the HNU15 slurry exhibited pH values of 9.11 to 9.20, DLS d_50_ values of 111 nm to 118 nm, and zeta potentials of −53.60 mV to −52.46 mV over the four-week period. The corresponding ranges for the HC10 slurry were pH 8.43 to 8.50, DLS d_50_ 122 nm to 128 nm, and zeta potential −49.40 mV to −48.62 mV.

At 60 °C, the HNU15 slurry exhibited pH values of 9.11 to 9.13, DLS d_50_ values of 111 nm to 116 nm, and zeta potentials of −53.60 mV to −52.92 mV. The corresponding ranges for the HC10 slurry were pH 8.43 to 8.44, DLS d_50_ 122 nm to 127 nm, and zeta potential −49.40 mV to −48.72 mV.

Across both temperatures, the measured properties showed limited changes, and neither slurry exhibited a continuous increase in DLS d_50_ or a substantial loss of negative zeta potential. The HNU15 slurry maintained a smaller DLS d_50_ and a more negative zeta potential than the HC10 slurry throughout the tested period. These results indicate that the measured colloidal properties of both slurries remained generally stable under the evaluated four-week storage conditions.

### 3.9. Proposed Material Removal Mechanism of PAA-Containing Ceria Slurries in STI CMP

**Figure 17:**
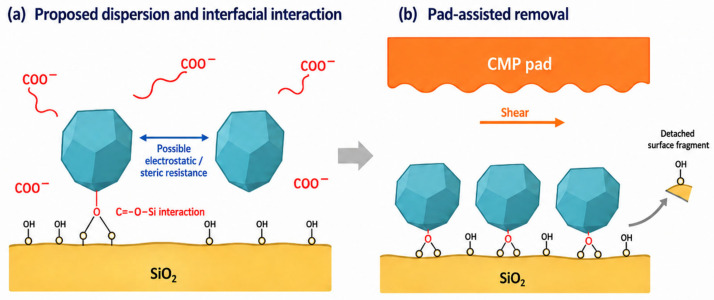
Literature-based proposed mechanism for STI CMP using a PAA-containing ceria slurry: (**a**) possible ceria–silica interfacial interaction and (**b**) pad-assisted removal and transport of reacted surface material.

Figure 17 presents a literature-based conceptual model for material removal by PAA-containing ceria slurries during the STI CMP process. According to the chemical-tooth model, hydroxylated ceria and silica surfaces may form interfacial Ce–O–Si species, after which mechanical shear assists the removal of reacted surface material [11,13,22]. The higher Ce^3+^ fraction observed for HNU15 indicates a difference in the ceria surface chemical state that has been associated with ceria–silica interactions in previous studies.

The PAA may influence particle–particle and particle–surface interactions through electrostatic and steric contributions at the ceria–water interface [43,44]. The measured DLS and zeta-potential values characterize the complete PAA-containing slurries but do not isolate a quantitative PAA effect because a PAA-free control was not included. Figure 17 should therefore be interpreted as a proposed mechanism consistent with established CMP and colloid concepts rather than as direct experimental demonstration of each interfacial step.

## 4. Conclusions

In this study, PAA was synthesized by aqueous free-radical polymerization and characterized by GPC and FT-IR. GPC analysis determined a number-average molecular weight (Mn) of 11,500 g mol^−1^, a weight-average molecular weight (Mw) of 21,300 g mol^−1^, and a polydispersity index of 1.85, while the FT-IR results supported the presence of carboxylic acid and carboxylate functionalities. The synthesized PAA was subsequently used at the same fixed loading as the polymeric dispersant in slurries prepared with HNU15 and HC10 ceria powders.

The primary-particle sizes determined by TEM were 12.2 ± 1.5 nm for HNU15 and 14.6 ± 1.4 nm for HC10, whereas the crystallite sizes calculated from XRD were 10.2 nm and 8.7 nm, respectively. BET analysis determined specific surface areas of 83.2 m^2^ g^−1^ and 60.2 m^2^ g^−1^ and average pore sizes of 5.42 nm and 6.14 nm for HNU15 and HC10, respectively. The Ce^3+^ fractions calculated from the fitted XPS spectra were 22.1% for HNU15 and 17.7% for HC10, demonstrating measurable differences in the surface chemical states of the two powders. These results characterize differences in textural and surface chemical properties but do not directly establish the density of active polishing sites or Ce–O–Si bond formation during CMP.

After 5 h of milling, the DLS d_50_ values decreased from 155 nm to 111 nm for the HNU15 slurry and from 160 nm to 122 nm for the HC10 slurry. The corresponding zeta potentials changed from −45.55 mV to −53.60 mV and from −43.33 mV to −49.40 mV, respectively. Both slurries maintained generally stable pH, DLS d_50_, and zeta potential during four weeks of storage at 25 °C and 60 °C.

Under the evaluated laboratory CMP conditions, the HNU15 slurry exhibited an HDP-SiO_2_ MRR of 114.4 Å min^−1^, an HDP-SiO_2_-to-Si_3_N_4_ selectivity of 8.0, and WIWNU values of 33% for HDP-SiO_2_ and 22% for Si_3_N_4_. It also produced lower post-polishing RMS roughness than the HC10 slurry. Because both slurries contained the same PAA loading and a PAA-free control was not included, the observed differences are interpreted at the complete-slurry level rather than being attributed solely to PAA. Overall, the combined polymer, colloidal, storage-stability, and CMP results support further optimization of the PAA-containing HNU15 slurry for STI CMP applications.

## Figures and Tables

**Table 1:** Relative integrated-intensity percentages of the indexed CeO_2_ reflections for commercial HC10 and prepared HNU15.

Crystal Plane (*hkl*)	Commercial HC10 (%)	Prepared HNU15 (%)
(111)	42.5	41.2
(200)	11.2	12.0
(220)	21.0	21.5
(311)	13.5	13.8
(222)	2.0	2.1
(400)	2.2	2.3
(331)	3.8	3.5
(420)	2.0	1.8
(422)	1.8	1.8
Total	100.0	100.0

**Table 2:** Primary-particle size, crystallite size, BET surface area, and average pore size of prepared HNU15 and commercial HC10 ceria powders.

Sample	*d*_TEM_ (nm)	*d*_XRD_ (nm)	BET Surface Area (m^2^ g^−1^)	Average Pore Size (nm)
Prepared HNU15	12.2 ± 1.5	10.2	83.2	5.42
Commercial HC10	14.6 ± 1.4	8.7	60.2	6.14

**Table 3:** Ce oxidation-state fractions and the high-binding-energy O 1s component of the prepared HNU15 and commercial HC10 ceria powders.

Sample	Ce^3+^ Fraction (%)	Ce^4+^ Fraction (%)	High-Binding-Energy O 1s Component (eV)
Prepared HNU15	22.1	77.9	533.05
Commercial HC10	17.7	82.3	533.20

Ce^3+^ and Ce^4+^ fractions were calculated from the integrated areas of the fitted Ce 3d components. The reported O 1s binding energies correspond to the higher-binding-energy component associated with surface or adsorbed oxygen species.

**Table 4:** Molecular-weight characteristics of the synthesized PAA determined by GPC.

Sample Name	Mn (g mol^−1^)	Mw (g mol^−1^)	PDI (Mw/Mn)
Prepared PAA	11,500	21,300	1.85

**Table 5:** Milling-dependent pH, DLS d_50_, and zeta potential values of the PAA-containing HNU15 and HC10 slurries.

Time (h)	Commercial HC10			Prepared HNU15		
	pH	DLS d_50_ (nm)	Zeta Potential (mV)	pH	DLS d_50_ (nm)	Zeta Potential (mV)
0	7.52	160	−43.33	8.51	155	−45.55
1	7.70	154	−44.28	8.62	151	−46.37
2	7.66	148	−44.10	8.58	145	−46.21
3	7.89	142	−46.12	8.80	138	−48.96
4	8.18	133	−47.85	8.97	124	−51.43
5	8.43	122	−49.40	9.11	111	−53.60

## Data Availability

The data presented in this study are available within the article.
